# Epigenetic Modifications in Peripheral Blood as Potential Noninvasive Biomarker of Diabetic Retinopathy

**DOI:** 10.1167/tvst.8.6.43

**Published:** 2019-12-18

**Authors:** Arul J. Duraisamy, Rakesh Radhakrishnan, Berhane Seyoum, Gary W. Abrams, Renu A. Kowluru

**Affiliations:** 1 Wayne State University, Department of Ophthalmology, Visual and Anatomical Sciences, Detroit, MI, USA; 2 PerkinElmer Health Sciences Pvt Ltd., Tharamani, India; 3 Wayne State University, Endocrinology, Detroit, MI, USA

**Keywords:** biomarker, diabetic retinopathy, DNA methylation, epigenetics, mitochondrial DNA

## Abstract

**Purpose:**

Progression of diabetic retinopathy is related to the duration and severity of hyperglycemia, and after 25 years of diabetes, 90% of patients show some signs of retinopathy. Despite initiation of many retinal molecular/biochemical abnormalities, including mitochondrial damage and epigenetic modifications, the disease remains asympotomatic in the initial stages. Our goal is to examine the utility of DNA methylation as a possible biomarker of diabetic retinopathy.

**Methods:**

Genomic DNA (gDNA) was isolated from the buffy coat, isolated from blood of diabetic patients with proliferative (PDR) or no retinopathy (No-DR), and nondiabetic subjects (CONT). Methylation of mitochondrial DNA (mtDNA), especially its *D-Loop* (the site of mtDNA transcription/replication), was quantified by methylated DNA immunoprecipitation and methyl-specific PCR techniques. Results were confirmed in purified mtDNA. The specific *D-Loop* region with the highest DNA methylation was identified using five overlapping primers, and DNMT1 binding was quantified by chromatin immunoprecipitation. Promoter DNA methylation of DNA mismatch repair (*MLH1*) and superoxide scavenging (*SOD2*) enzymes were also quantified.

**Results:**

Compared to CONT, *D-Loop* methylation was higher in PDR and No-DR groups, and the *D-Loop* region responsible for encoding the majority of the mtDNA-encoded genes had significantly higher methylation in the PDR group versus No-DR. Similarly, compared to No-DR, the PDR group also had hypermethylated *MHL1* and *SOD2* promoters.

**Conclusions:**

Blood from PDR patients have higher DNA methylation, than seen in diabetic patients without retinopathy. Thus, DNA methylation can be used as a possible biomarker of diabetic retinopathy.

**Translational Relevance:**

DNA methylation status in the blood of diabetic patients could serve as a potential noninvasive biomarker of retinopathy, and also an important readout parameter for testing longitudinal outcome of novel therapeutics for this blinding disease.

## Introduction

The International Diabetes Federation estimates approximately 425 million adults had diabetes in 2017, and with the prevalence of this disease rising at an alarming rate, by 2040 this number could be 642 million.^1^ In the eye, sustained circulating high blood glucose damages the retina and its vasculature, and diabetic retinopathy is considered as the leading cause of acquired blindness in working-age adults.^2^–^4^ This blinding disease is a progressive disease that affects about 90% of patients after 25 years of diabetes, and compared to type 2 diabetic patients, its prevalence is higher in those with type 1.^5^

Although diabetic retinopathy has a long asymptomatic phase, it can progress from the moderate to the severe nonproliferative stage and then to the proliferative stage in a relatively short period.^2^,^6^ Severity of hyperglycemia is considered the key alterable risk factor, with the duration of diabetes also intimately related to the development and progression of retinopathy. Although maintenance of tight glycemic control impedes the development of diabetic retinopathy, in many patients, especially those with type 2 diabetes, hyperglycemia may remain undiagnosed for a long period of time.^3^,^7^–^9^ Also, the majority of patients do not, or cannot, maintain tight glycemic control for long durations, and their retina continues to be exposed to a hyperglycemic insult. Thus, identifying noninvasive biomarkers to foretell the development of diabetic retinopathy and/or access the outcome of therapeutic intervention is critical.

The pathogenesis of diabetic retinopathy is complex, involving interrelated metabolic, functional, and structural abnormalities. Novel technical advances have identified many metabolic abnormalities associated with diabetic retinopathy, however, the exact mechanism of the development of this sight-threatening disease remains unclear.^2^,^10^ Hyperglycemia increases reactive oxygen species and damages the retinal mitochondrial integrity, and the defense mechanism to repair damaged mitochondria is impaired. Furthermore, mitochondrial DNA (mtDNA) is also damaged, compromising the electron transport chain system.^10^–^14^

Hyperglycemic environment also alters the transcripts of many genes, and recent research has documented the involvement of external factors, including environmental factors and lifestyle, in gene expression and disease progression.^15^,^16^ These epigenetic modifications change the gene expression (and the phenotype) without affecting the underlying DNA sequence, and many epigenetic modifications have now been recognized to play a significant role in the development of diabetes and its complications.^17^,^18^

Methylation of cytosine in the DNA forms methyl cytosine (5mC); about 1.5% of the human genomic DNA (gDNA) has 5mC, and hypermethylation of a gene promoter is associated with its repression.^19^ DNA methylation is mediated by DNA methyl transferases (DNMTs), and among this family of DNMTs, DNMT1 appears to be responsible for the maintenance of established patterns of DNA methylation and DNMT3a and 3b for establishment of de novo DNA methylation patterns.^20^,^21^ Methylated cytosine can be hydroxymethylated by the 10-11 translocation (TET) family of enzymes, forming 5 hydroxymethyl cytosine (5hmC), which results in transcriptional activation.^22^,^23^ Many potential CpG sites are reported to undergo DNA methylation in the blood from type 1 diabetic patients,^24^ and compared to patients with normal albumin level, diabetic patients with albuminuria have higher global DNA methylation.^25^ Furthermore, several epigenetic modifications have also been shown to modify genes associated with metabolic abnormities implicated in the development of diabetic retinopathy.^10^,^26^,^27^

Mammalian mitochondria contain a small circular DNA (∼16 kbp), which is critical for encoding genes for 13 proteins that are essential in the functioning of the electron transport chain.^28^,^29^ Separating the strands of mtDNA is a loop-like noncoding region, the displacement loop (*D-Loop*), located between 16024 and 576 base pairs, which is the major site of initiation of transcription and replication.^30^–^32^ In diabetic retinopathy, retinal mtDNA is oxidatively modified and sequence variants are increased, and the damage is more extensive at the *D-Loop* compared to the other regions of mtDNA.^33^

Mitochondrial DNA is less than 1% of the total cellular DNA, but it has approximately 450 CpG sites and 4500 cytosine at non-CpG sites.^34^ Mitochondria are also equipped with DNA methylation machinery; DNMT1 has a mitochondrial targeting sequence,^35^ and TET is also reported in the mitochondria.^36^ Methylation of mtDNA is associated with decreased transcriptional capacity of proteins encoded by mtDNA and is observed in many mtDNA-related chronic diseases,^35^–^38^ and increased mtDNA 5hmC levels are observed after ischemia/reperfusion injury.^39^ Since the establishment and maintenance of epigenetic modifications are mediated by external factors, and these modifications can either be erased or passed to the next generation,^15^ epigenetic modifications have potential to serve as good candidates for disease biomarkers.

Mitochondria play a central role in the development of diabetic retinopathy, and changes in mtDNA affect their cellular function.^10^,^40^,^41^ In addition, methylation *D-Loop*, via compromising mtDNA transcription, further compromises the electron transport chain system and damages mitochondrial homeostasis.^42^ Higher blood mtDNA methylation levels are related with increased susceptibility to air pollution–induced heart rate variability,^43^ and mitochondrial epigenetic modifications are linked to increased risk of diabetes.^44^ The methylation status of blood mtDNA in diabetic patients, however, is not clear.

Our study using a limited number of patients with documented diabetic retinopathy, and animal models of diabetic retinopathy have shown a direct relation between mtDNA damage in the peripheral blood and diabetic retinopathy.^45^ The goal of our present study is to examine mtDNA methylation in the peripheral blood of diabetic patients with proliferative retinopathy or no retinopathy and to determine the utility of DNA methylation as a possible biomarker of diabetic retinopathy.

## Methods

### Human Subjects

Diabetic patients, both male and female, visiting the Endocrinology Clinic at the Detroit Medical Center (Detroit, MI) were approached for the study. After receiving a written informed consent, blood (∼5 mL) was collected in ethylene diamine tetra acetic acid vacutainers. Diabetes status was defined as hemoglobin A1c ≥ 6.5%. Based on the ophthalmic evaluation, diabetic patients were divided in two groups: group 1 patients had proliferative retinopathy (PDR, 23 patients), and group 2 patients had no diabetic retinopathy (No-DR, 23 patients). These patients had received their ophthalmic evaluation at the Kresge Eye Institute (Detroit, MI), within 6 months of their blood draw. Their retinopathy was graded using the International Clinical Disease Severity Scale for Diabetic Retinopathy. Healthy, nondiabetic subjects, without any retinal complication, were recruited as controls (CONT, 15 volunteers). Subjects with malignancy, trauma, or age under 18 were excluded from the study. The Wayne State University Institutional Review Board approved the protocol, and it complied with all aspects of the Health Insurance Portability and Accountability Act and was conducted in accordance with the tenets of the Declaration of Helsinki.

### Genomic DNA

Blood was centrifuged at 800 × *g* for 10 minutes, and the concentrated leukocyte suspension (buffy coat) between the plasma and erythrocytes was removed in a fresh tube.^46^ Genomic DNA was isolated from the buffy coat using a DNA purification kit (QIAamp DNA Blood Mini Kit, Cat No: 51104; Qiagen, Valencia, CA). Briefly, the DNA was loaded on the silica gel membrane columns, and after washing the columns to remove the impurities, the bound DNA was eluted using the elution buffer provided with the kit. The concentration and purity of DNA was quantified in a plate reader at 260/280 nm.

### DNA Methylation

The levels of 5mC and 5hmC were quantified by using methylated and hydroxymethylated DNA immunoprecipitation kits (MeDIP and hMeDIP kits respectively, Cat. No. P-1015, P-1038; EpiGentek, Farmingdale, NY). Using 250 ng gDNA for 5mC and 500 ng for 5hmC, DNA bound to the high DNA affinity strip-wells was captured using 5mC or 5hmC antibody. Enriched fractions of 5mC/5hmC were analyzed by quantitative real-time PCR (qPCR) using specific primers, and relative fold change in 5mC concentration was calculated using the delta Ct (ddCt) method.^40^,^47^

Methylation was also analyzed using methyl-specific PCR (MSPCR) by employing a kit (Epitect Plus Lyse All Bisulfite kit, Cat. No. 59124; Qiagen). Using 1 μL of bisulfite-converted gDNA as the template, 4 μL GoTaq reaction buffer, 0.8 mM dNTPs, GoTaq DNA polymerase, and 0.5 μM of the primer pairs specific for methylated and unmethylated sequences, qPCR was performed (Table 1). The amplified PCR products were resolved on a 2% agarose gel, and the ratio of band intensities of methylated to unmethylated was analyzed using ImageJ software (http://imagej.nih.gov/ij/; provided in the public domain by the National Institutes of Health, Bethesda, MD), as reported previously.^40^

**Table 1:** Primer Sequences

Primer	Sequence (5′–3′)
*D-Loop*	ATGGGGAAGCAGATTTGGGT
	GCGTTTTGAGCTGCATTGCT
Methylated *D-Loop*	TAAGAGTGTTATTTTTTTCGTTTCG
	ATAAAATACTCCGACTCCAACGTC
Unmethylated *D-Loop*	TAAGAGTGTTATTTTTTTTGTTTTGG
	CATAAAATACTCCAACTCCAACATC
*DNMT1*	AGTCCGATGGAGAGGCTAAG
	TCCTGAGGTTTCCGTTTGGC
*TET2*	TGGATTGCTGCAAGGCTGAG
	CTCAACAGGAGCAAAGGCAAG
*COXII*	GGCACATGCAGCGCAAGTAGG
	GGCGGGCAGGATAGTTCAGAC
*ND1*	ACGGGCTACTACAACCCTTC
	ATGGTAGATGTGGCGGGTTT
*CYTB*	TCACCAGACGCCTCAACCGC
	GCCTCGCCCGATGTGTAGGA
*B ACTIN*	AGCCTCGCCTTTGCCGATCCG
	TCTCTTGCTCTGGGCCTCGTCG
*MLH1* promoter	CCTGGCTCGGTTAAAAAGC
	GGAGGTGTTGCTGAGAGAGG
*SOD2*	CCTGCTCCCCGCGCTTTCTT
	CGGGGAGGCTGTGCTTCTGC
*SOD2* promoter	ATACGGGTTGGAAGGGCGCTG
	TGAGTTTTGGTTGCGCTGCCG
*MLH1*	TATGGCTTTCGAGGTGAGGC
	CCTTGATTGCCAGCACATGG

To confirm methylation, an endonuclease digestion method was also employed. Briefly, gDNA (2 μg) was subjected to restriction digestion by the methylation-sensitive *Hpa*II restriction enzyme (the enzyme that cleaves only unmethylated -CCGG- consensus sequences) in a 20 μL reaction mixture containing 2 μL of cutsmart buffer.^33^ The reaction was terminated by incubating the mixture at 90°C, and the digested DNA was amplified for *D-Loop* using primers flanking the restriction sites (Table 1). Increased DNA methylation reciprocate with higher target amplification due to reduced *Hpa*II activity and vice versa.

To rule out the possibility of contamination of nuclear DNA, 5mC levels were also quantified in the purified mtDNA in five random samples per group. Mitochondrial DNA was purified using a purification kit (K389-25; BioVision, Milpitas, CA), and 5mC levels were measured using EpiQuik methylated DNA immunoprecipitation kit (EpiGentek).

To further investigate the pattern of DNA methylation, five different sets of overlapping primers of *D-Loop* were employed. The selection of these regions was based on the CpG density (Table 2).

**Table 2:** Primer Sequences of Five Regions of the D-Loop

Region	Sequence (5′–3′)	Spanning Region	Product Length	CpGs
Region 1	ACATTACTGCCAGCCACCAT	16098–16276	179	1
	ATCCTAGTGGGTGAGGGGTG			
Region 2	CACCCCTCACCCACTAGGAT	16257–16410	154	2
	GAGGATGGTGGTCAAGGGAC			
Region 3	GTCCCTTGACCACCATCCTC	16391–16549	159	6
	GGGGAACGTGTGGGCTATTT			
Region 4	ACATCTCTGCCAAACCCCAA	340–502	163	2
	GGCGGGGGTTGTATTGATGA			
Region 5	TCATCAATACAACCCCCGCC	483–638	157	4
	GGTGATGTGAGCCCGTCTAA			

### DNMT1 Binding

Chromatin immunoprecipitation (ChIP) technique was employed to quantify the binding of DNMT1 and TET2 at the *D-Loop*. Briefly, the buffy coat (for DNMT1) was crosslinked with 1% paraformaldehyde; the crosslinked protein-DNA complex (120 μg) was immunoprecipitated with 3 μg DNMT1 (Cat. No. ab13537; Abcam, Cambridge, MA) and captured using protein-A agarose beads. Samples were then washed, de-crosslinked at 65°C, and digested with proteinase K. The DNA was purified using the phenol-chloroform method and was amplified for *D-Loop* using the specific primers (Table 1). Input DNA (40 μg) was used as an internal control, and 3 μg IgG was used (Cat. No. ab171870; Abcam) as an antibody control.^40^,^48^

Sequence mismatches in the *D-Loop* were analyzed using a detection kit (Surveyor Mutation Detection Kit; Integrated DNA Technologies, Coralville, IA) as described previously.^48^ Briefly, *D-Loop* was amplified from the DNA using specific primers (Table 1) and 1 U high-fidelity enzyme mixture (Elongase, Cat. No. 104800; Thermo Fisher Scientific, Waltham, MA) with an initial incubation cycle of 1 minute at 75°C, 1 minute at 94°C, and 24 cycles of 15 seconds at 94°C and 12 minutes at 65°C. The final extension was performed for 10 minutes at 72°C. The amplicon was digested using surveyor nuclease and a mismatch-specific endonuclease, and the digested products were resolved on 2% agarose gel. The gel was analyzed for fragmentation using a UV transilluminator, and the amplicon band intensity was quantified using ImageJ.

### Gene Transcripts

Total RNA from buffy coat was isolated by Trizol reagent (Cat No. 15596018; Invitrogen, Carlsbad, CA) and was converted to cDNA using a reverse transcription kit (High Capacity cDNA Reverse Transcription Kit, Cat No. 4368814; Applied Biosystems, Foster City, CA). Using a master mix (Power SYBR Green PCR Master Mix, Cat No. 4367659; Applied Biosystems) and gene-specific primers (Table 1), gene transcripts were quantified by qPCR. The fold change was calculated using ddCt method.^45^

Statistical analysis was performed using statistical software (Sigma Stat; Systat Software, San Jose, CA). One-way ANOVA followed by Student-Newman-Keuls test was used to compare between groups with normal distribution. Kruskal-Wallis one-way analysis followed by Dunn's test was used to compare between groups with nonnormal distribution. Data are expressed as mean ± SD, and *P* < 0.05 was considered statistically significant.

## Results

Both PDR and No-DR groups had age-matched patient population (median age 53.5 years, range, 33–75 years, and 53.7 years and 30–77 years, respectively). The PDR group had 46% male patients and the No-DR group had 43%. Despite differences in the number of type 1 diabetic patients (39% in PDR group compared to 13% in No-DR group) and relatively longer median duration of diabetes in PDR group (6–54 years versus 1–24 years), their HbA1_C_ values were similar (7%–11% PDR versus 6.5%–13% No-DR). The CONT group had 36% male subjects, and compared to PDR and No-DR groups, the CONT group had slightly younger individuals (median age 45 years, range 30–66 years). However, the age differences among the three groups was not statistically significant (*P* = 0.08 CONT versus PDR and *P* = 0.31 CONT versus No-DR).

**Figure 1:**
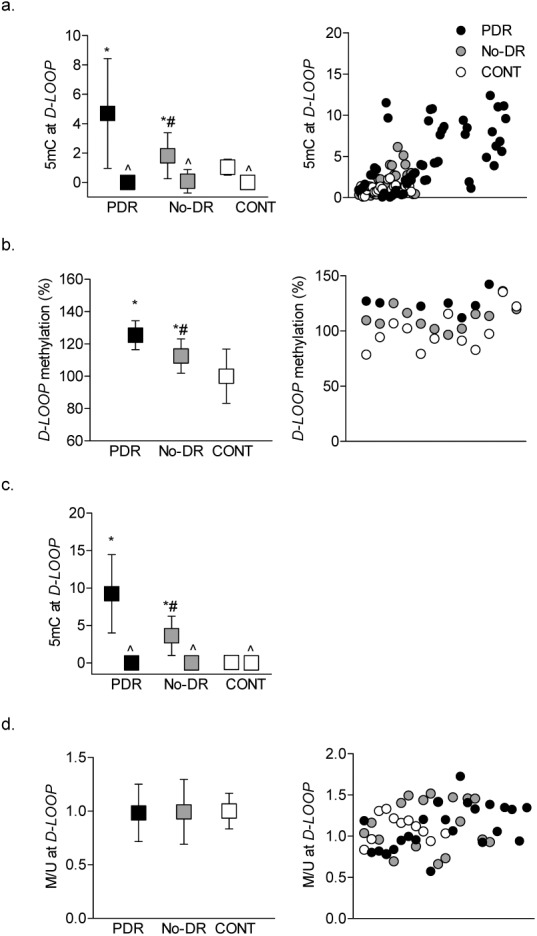
Methylation of mtDNA in the peripheral blood. Cytosine methylation in the D-Loop was quantified in the buffy coat (a) by a methylated DNA (MeDIP) immunoprecipitation kit. Fold change values obtained from qPCR were quantified by ddCt method and (b) by digesting DNA with methylation-sensitive HpaII endonuclease followed by qPCR using specific primers spanning the HpaII restriction site (-CCGG-). (c) The 5mC levels were quantified in purified mtDNA by MeDIP immunoprecipitation method. (d) Methylation-specific PCR was performed in the bisulfite-converted DNA, and the ratio of the methylated (M) to unmethylated (U) band was plotted. Value obtained from nondiabetic subjects was considered as one. The data are presented as mean ± SD obtained from 12 to 25 diabetic patients, each with proliferative retinopathy (PDR) or without retinopathy (No-DR), and nondiabetic control subjects (CONT). *,#P < 0.05 versus CONT and PDR groups respectively; ^IgG control antibody.

Peripheral blood from diabetic patients has higher *D-Loop* damage compared to nondiabetic controls, and the damage is significantly higher in patients with proliferative retinopathy compared to patients without retinopathy.^4^ Consistent with this, 5mC levels at the *D-Loop* were approximately two-fold higher in the PDR group compared to the No-DR group (Fig. 1a); values obtained using IgG antibody control were <0.01% of the values obtained from 5mC antibody. In accordance with 5mC levels, 20% to 40% higher DNA methylation was observed in the PDR group by methylation-specific endonuclease digestion methods, and the values were significantly different from the values obtained in CONT group (Fig. 1b). Consistent with the results from the gDNA, compared to CONT group 5mC levels at the *D-Loop* were significantly higher in the purified mtDNA in both PDR and No-DR groups, and among these two diabetic groups, the PDR group had approximatley three-fold higher 5mC levels compared to the No-DR group (Fig. 1c). Furthermore, our preliminary results from bisulfite DNA sequencing^44^,^45^ in a very limited number of samples (two to three samples per group) also showed similar differential *D-Loop* cytosine methylation. However, in contrast to the results from endonuclease digestion and immunoprecipitation methods, MSPCR showed similar ratios of methylated:unmethylated cytosine in all three groups (Fig. 1d).

**Figure 2:**
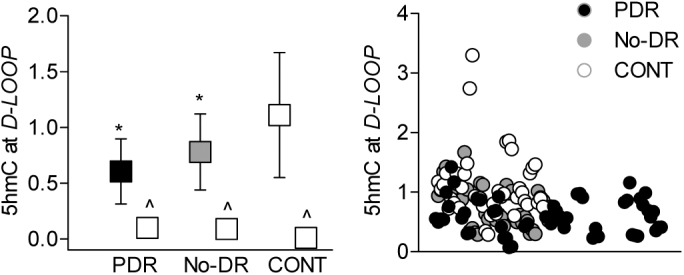
Hydroxymethylation of mtDNA. (a) The levels of 5hmC were quantified in human blood buffy coat using a hMeDIP immunoprecipitation kit. The values are represented as mean ± SD obtained from 15 to 20 patients in each of the three groups. *,#P < 0.05 compared to CONT and PDR, respectively.

Since MSPCR and bisulfite DNA sequencing methods do not discriminate between 5mC and 5hmC,^37^ to further confirm differences in DNA methylation, 5hmC levels were quantified. As shown in Figure 2, compared to CONT group, both PDR and No-DR groups had similar 5hmC levels, but these values were significantly lower compared to those obtained from the CONT group. To confirm the role of TET2 in the methylation status of *D-Loop*, TET2 binding was quantified in the mitochondria isolated (Mitochondria Isolation Kit 89874; ThermoScientific) from the buffy coat from two samples each in CONT and No-DR groups. TET2 binding was relatively lower in the No-DR group compared to the CONT group (1.14 versus 1.42), confirming mitochondrial localization of TET2 and its role in maintaining methylation status of mtDNA. However, due to limitation of the samples, we were unable to analyze statistical significance between these two groups and perform TET2 binding in the PDR group.

**Figure 3:**
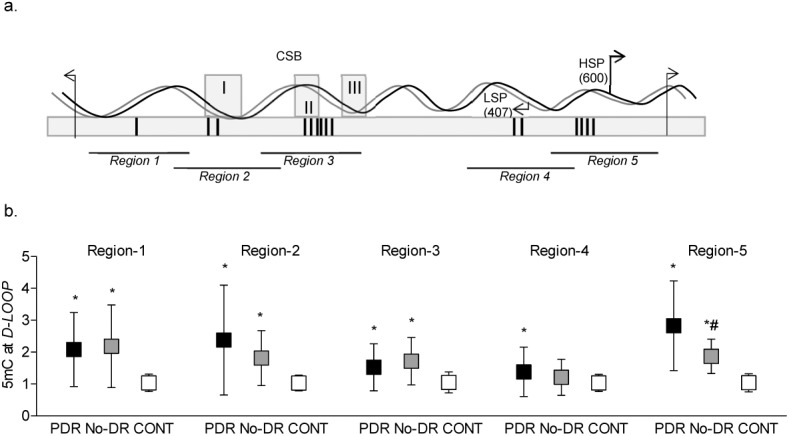
DNA methylation of different CpG-rich regions of the D-Loop. (a) Schematic diagram of five regions of the D-Loop (16024-576 bp region) with differential CpG density. Vertical bars in the box represent CpG density; 1, 2, 6, 2, and 4 CpG sites, respectively. Regions 2 and 3 also cover three conserved sequence box domains (CSB-I, II, and III), and region 4 and 5 have light strand promoter (LSP) and heavy strand promoter (HSP), respectively. (b) 5mC levels in the regions 1 to 5 of the D-Loop were quantified in immunoprecipitated samples using a MeDIP kit. *,#P < 0.05 versus CONT and PDR groups, respectively.

To further investigate cytosine methylation and its distribution pattern in the *D-Loop*, five different overlapping regions of the *D-Loop* (Fig. 3a), compared to the CONT, PDR, and No-DR groups, had increased 5mC levels in all of the five regions. However, compared to the No-DR group, the PDR group had significantly higher (>80%) 5mC levels in region 5. In addition, although region 2 had ∼40% higher 5mC in the PDR group, these values were not statistically significant compared to the values obtained in the No-DR group (Fig. 3b).

**Figure 4:**
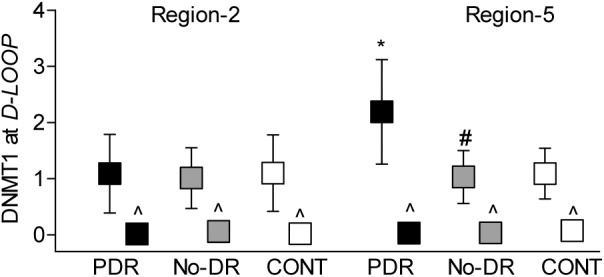
DNMT1 binding at the D-Loop. Regions 2 and 5 of the D-Loop were analyzed for DNMT1 binding by ChIP technique; IgG was used as an antibody control (^). The values are represented as mean ± SD. *P < 0.05 versus CONT and #P < 0.05 versus PDR.

To confirm differences in DNA methylation in these regions, the binding of DNMT1 was investigated in regions 2 and 5. Compared to the No-DR group, the PDR group had approximately two-fold higher DNMT1 binding in region 5, but in region 2, DNMT1 binding was similar in the PDR, No-DR, and CONT groups; IgG control antibody yielded values that were <0.01% of the values obtained from DNMT1 antibody (Fig. 4).

**Figure 5:**
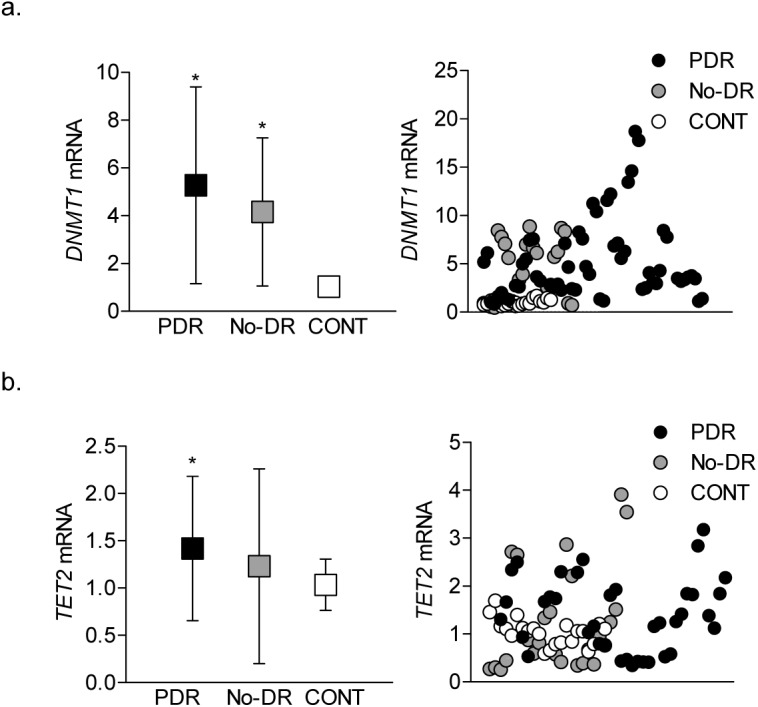
DNA methylation-hydroxymethylation machinery. Transcript levels of (a) DNMT1 and (b) TET2 were quantified in the cDNA by qPCR using β-ACTIN as the housekeeping gene. The data are mean ± SD from 15 to 20 samples in each of the three groups (PDR, No-DR, and CONT). *P < 0.05 compared to the CONT group.

Methylation of cytosine is mediated by DNMTs, but 5mC can also be rapidly hydroxymethylated by TETs.^35^ We quantified the gene transcripts of DNMTs and TETs. Compared to the CONT group, DNMT1 was elevated by four- to five-fold in both the PDR and No-DR groups (Fig. 5a). However, although TET2 was also elevated in both the PDR and No-DR groups, the values in the No-DR group did not achieve any statistical significance (Fig. 5b).

**Figure 6:**
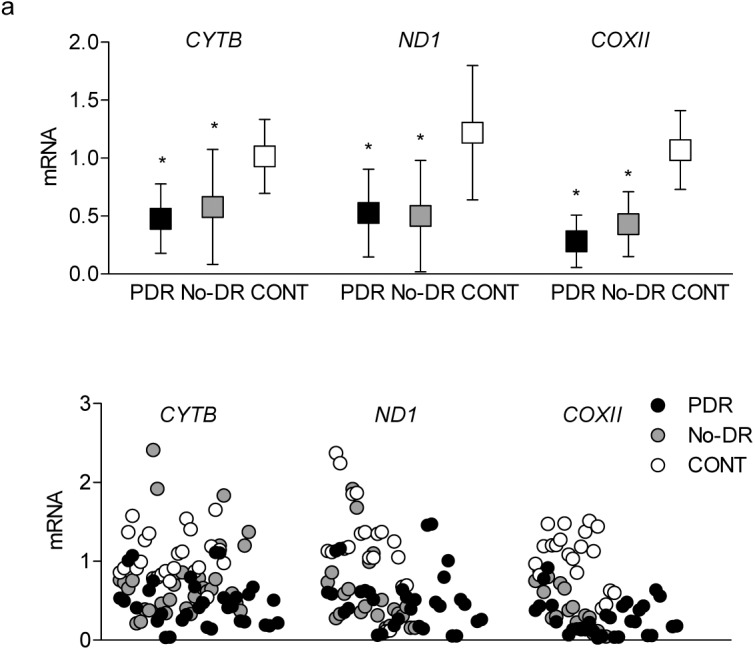
Transcripts of mtDNA-encoded genes. Gene transcripts of CYTB, ND1, and COXII were quantified in the cDNA using qPCR, and the data obtained were normalized against the values from β-ACTIN by the ddCt method. *P < 0.05 versus CONT.

Since methylation of DNA impairs its transcriptional capacity,^10^,^35^ expression of mtDNA-encoded genes was measured in the cDNA prepared from the peripheral blood. Consistent with hypermethylation of mtDNA, expressions of NADH-ubiquinone oxidoreductase chain 1 (*ND1*) of complex I, cytochrome b (*CYTB*) of complex III, and cytochrome oxidase II (*COXII*) of complex IV were significantly decreased in both the PDR and No-DR groups as compared to the values obtained from the CONT group (Fig. 6).

**Figure 7:**
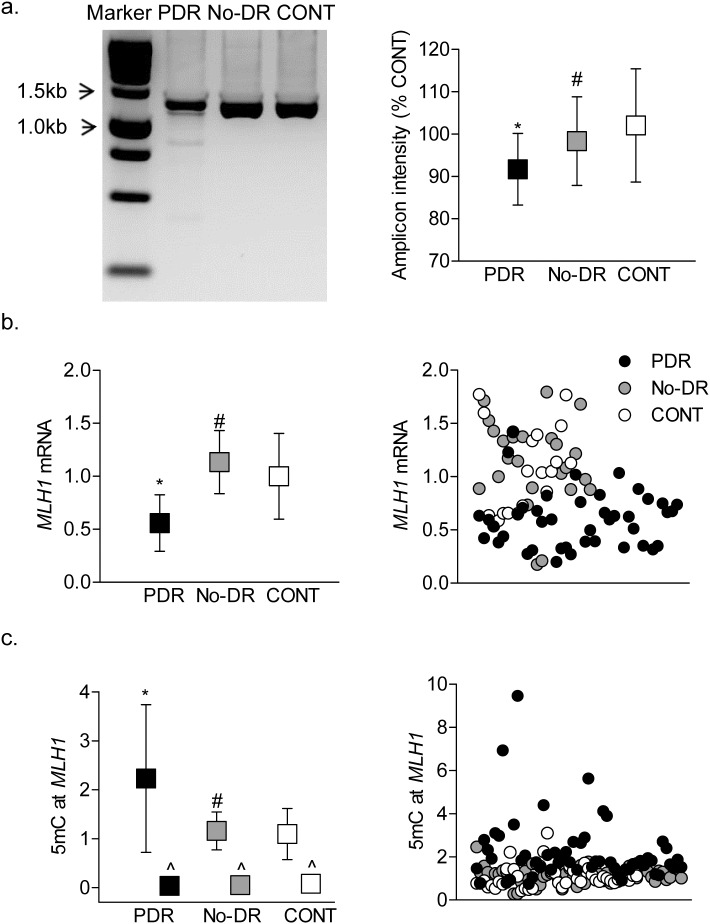
Mismatches in the D-Loop. (a) Base mismatches in the D-Loop were analyzed after digesting the complete D-Loop amplicons with mismatch-specific surveyor nuclease, followed by resolving in agarose gel for fragmentation analysis and quantification of the amplicon intensity. The intensity of the amplicon in the CONT group was considered as 100%. (b) Gene transcripts of MLH1 were quantified by qPCR using β-ACTIN as a housekeeping gene. (c) 5mC levels in the MLH1 promoter were quantified by methylated DNA immunoprecipitation technique using IgG as an antibody control (^). The data are mean ± SD obtained from 12 to 20 subjects in each group. *P < 0.05 versus CONT and #P < 0.05 versus PDR.

DNA methylation can also result in increased base mismatches; in diabetes, increased mismatches are observed in the *D-Loop*, and the mismatch repair system is compromised in the retinal vasculature.^33^ Consistent with retinal vasculature, base mismatches were significantly higher in the PDR group compared to the No-DR group (Fig. 7a). The PDR group also had reduced gene transcripts *MLH1*, the enzyme responsible for repair of DNA mismatches, and the *MLH1* promoter DNA was also hypermethylated (Figs. 7b and c), suggesting the role of DNA methylation in decreased *MLH1* expression. However, in contrast to increased *D-Loop* methylation in the No-DR group, base mismatches, *MLH1* expression, and its promoter DNA methylation were similar to those observed in the CONT group.

**Figure 8:**
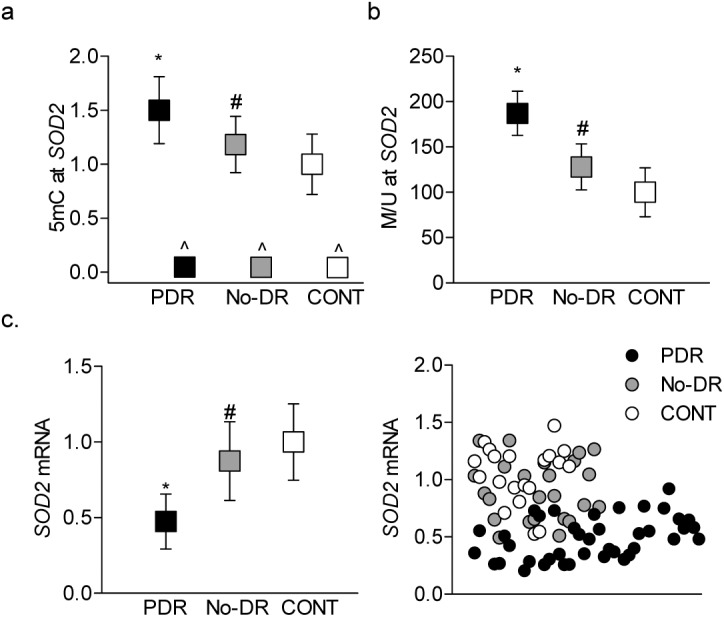
Superoxide scavenging enzyme in the buffy coat. DNA methylation of SOD2 promoter was determined by (a) quantifying 5mC levels using the methylated DNA immunoprecipitation technique, with IgG (^) included as an antibody control, and (b) by methylation-specific PCR. (c) Gene transcripts of SOD2 were quantified by qPCR using β-ACTIN a housekeeping gene. *P < 0.05 versus CONT and #P < 0.05 versus PDR.

Decreased transcription of mtDNA-encoded genes compromises the electron transport chain, resulting in increased mitochondrial superoxide levels, and manganese superoxide plays a critical role in scavenging mitochondrial superoxide radicals.^10^,^49^ To further investigate the possibility of using DNA methylation as a potential biomarker of diabetic retinopathy, DNA methylation of *SOD2*, the gene encoding manganese superoxide dismutase (MnSOD), was examined. Figure 8a shows that, compared to CONT group, 5mC levels at the *SOD2* promoter were significantly higher only in the PDR group; however, the No-DR group showed an insignificant change compared to CONT group, and 5mC, and its values are significantly lower than the PDR group. Results using the MSPCR method further confirmed hypermethylation of the *SOD2* promoter in the PDR group (Fig. 8b). In accordance with *SOD2* promoter hypermethylation, *SOD2* mRNA was decreased by 50% in the PDR group, and although in the No-DR group the decrease in *SOD2* mRNA was only 20%, these values were significantly different from those obtained from PDR group (Fig. 8c).

## Discussion

Diabetic retinopathy remains the major blinding disease in working-age adults. This progressive disease is multifactorial, and in the initial stages it is generally asymptomatic.^6^ The lag time between diagnosis of diabetes and detection of any signs of retinopathy may extend to 10 to 20 years; however, the biochemical, molecular, and physiological changes induced by the hyperglycemic milieu continue to manifest. Mild to moderate nonproliferative retinopathy may progress to the severe nonproliferative stage and can become proliferative in a relatively short time.^2^ This makes identification of early, noninvasive biomarkers essential to protect the retina from the progressive pathology and vision loss. Mitochondrial dysfunction is considered as one of the major contributors in the development of diabetic retinopathy. Retinal mitochondria are swollen, mtDNA is damaged, and the expression of mtDNA-encoded genes is decreased, resulting in an electron transport chain system functioning at less than its optimal capacity. To make a bad situation worse, the defense system to protect mitochondrial functional and DNA damage is also compromised, and the vicious cycle of free radicals continues to self-perpetuate.^10^–^12^,^26^ Our previous work using retinal microvasculature from both rodent models and a limited number of diabetic patients (with or without documented retinopathy) has demonstrated a correlation between mtDNA damage in the peripheral blood and diabetic retinopathy.^45^ Recent technical advancements have implicated epigenetic modifications in the development of diabetic complications, including retinopathy,^10^,^26^,^50^ and epigenetic modifications in mtDNA are also observed in many diseases, including diabetic retinopathy.^33^,^37^,^40^ Here, we demonstrate that methylation of mtDNA is different in the peripheral blood from diabetic patients with retinopathy and without retinopathy. Also, we show convincing differences in the DNA methylation of the genes encoding mismatch repair enzyme *MLH1* and mitochondrial superoxide scavenging enzyme *SOD2*. The results suggest that DNA methylation status in the peripheral blood could potentially predict the progression of diabetic retinopathy to the proliferative stage and serve as a noninvasive biomarker.

Gene expression is influenced by external factors and disease state, and the chemical tags added to the chromosomes switch genes on or off without altering the DNA sequence.^15^,^16^ These epigenetic modifications can be erased or passed to the next generation. One of the major epigenetic modifications is DNA methylation in which covalent addition of a methyl group at the 5-carbon of the cytosine ring results in formation of 5mC, and presence of 5mC in the promoter region is associated with long-term transcriptional silencing.^20^,^21^ The CpG-rich circular mtDNA lacks protective histones and is a good target for methylation. In the pathogenesis of diabetic retinopathy, retinal mtDNA is hypermethylated, and DNMTs are activated. This hypermethylation is more extensive at the *D-Loop*, the region of mtDNA that is the major control site for mtDNA transcription and replication.^10^,^12^,^14^,^26^,^33^ The *D-Loop* is also the point of attachment of mtDNA to the mitochondrial inner membrane, and it is more prone to damage than other regions of the mtDNA.^51^ Here, using two independent methods, we show that compared to the CONT group, peripheral blood from both the PDR and No-DR groups have higher methylation in the *D-Loop*, and methylation is significantly higher in the PDR group compared to the No-DR group.

DNA methylation is a dynamic process, and 5mC can be hydroxymethylated to 5hmC^23^,^35^; we show that diabetic groups have lower 5hmC levels than does the CONT group. This suggests that our failure to observe increased *D-Loop* DNA methylation in the same samples by MSPCR technique could be due to the inability of this technique to distinguish between 5mC and 5hmC. In accordance with this, the decrease in 5hmC in the PDR group is accompanied by over a four-fold increase in *DNMT1* and about a 1.5-fold increase in *TET2*, suggesting that methylation of cytosine could be overpowering its hydroxymethylation.

*D-Loop* contains regulatory elements involved in mtDNA replication and transcription and has two hypervariable regions.^32^ It also has two promoters for RNA transcription: one promoter controls transcription of the H-strand (HSP), and the other controls transcription of the L-strand (LSP) where the transcription factors mtTFA and mtTFB bind to specific sequences upstream of both the HSP and LSP.^51^,^52^ Analysis of the different regions of the *D-Loop* in the blood shows hypermethylation of all of these regions in the PDR and the No-DR groups. Among these, however, region 5 has significantly higher methylation in the PDR group compared to the No-DR group. Consistent with this, compared to the other four regions of the *D-Loop*, region 5 has significantly higher DNMT1 binding in the PDR group. Interestingly, region 5 is the site of the promoter that controls the transcription of *HSP* and is responsible for encoding 28 out of 37 mtDNA-encoded genes.^53^ Methylated cytosine in the promoter region of the *HSP* is also implicated in the processing of RNA primer during replication of the H-strand,^19^ further supporting the importance of mtDNA methylation in regulating mitochondrial functioning. However, despite similar DNMT1 binding in region 2 in the three groups, the DR and No-DR groups have increased 5mC levels; DNA methylation is a dynamic process where TETs can convert 5mC to 5hmC,^47^ and the possibility of suboptimal conversion of 5mC to 5hmC in this region cannot be ruled out.

Methylation of cytosine is a natural phenomenon, and although both cytosine and methylated cytosine can be deaminated to thymine, deamination rate of methylated cytosine is several fold higher than that of cytosine.^54^ Moreover, mtDNA is located closer to the electron transport chain and has a 1000-fold higher mutation rate than does the nuclear DNA.^35^ Mitochondria also have an efficient mismatch repair systems to target replication errors that escape proofreading, and MLH1 is one of the major enzymes responsible for mismatch repairs in mtDNA.^48^,^55^ The results show major differences in the methylation status of *MLH1* promoter, and while peripheral blood from the No-DR and CONT groups have similar 5mC levels at the *MLH1* promoter, 5mC levels are significantly elevated in the PDR group.

Damage to mtDNA impairs its transcription and compromises the electron transport chain system, leading to increased superoxide radicals.^10^,^26^ Retinal microvasculature from diabetic rodents and human donors with diabetic retinopathy have reduced levels of mtDNA-encoded genes, including *ND1*, *CYTB*, and *COXII*.^56^ Although the levels of these genes are decreased significantly in the blood of diabetic patients with or without proliferative retinopathy, our results show that the decrease in *CYTB*, the only mtDNA-encoded protein of the complex III, is significantly higher in PDR group compared to the No-DR group.

Mitochondrial matrix has appreciable levels of MnSOD to scavenge free radicals, and decrease in MnSOD increases the steady-state levels of free radicals.^57^ Here, the results show that the transcripts of *SOD2* are significantly decreased in both the PDR and No-DR groups, and the decrease is significantly greater in the PDR group compared to the No-DR group. The transcription of *SOD2* is regulated by many regulatory regions, including a GC-rich core promoter adjacent to the transcriptional start site and an enhancer in the second intron.^58^ Consistent with decrease in *SOD2* transcripts, diabetic patients have hypermethylated *SOD2* promoter DNA, and compared to the No-DR group, DNA methylation is higher in the PDR group, further supporting the utility of peripheral blood DNA methylation as a possible biomarker of diabetic retinopathy.

We recognize that our study has some limitations, including 21% fewer samples in the CONT group compared to the PDR and No-DR groups. The CONT group also had slightly younger individuals, with 45 years median age compared to about 54 years median age in the PDR and No-DR groups. The gender of the patient is a factor in the severity of diabetic retinopathy. In male patients, advanced retinopathy is more common, and they have more abnormal local neuroretinal function compared with females^59^; although distribution of males and females in the PDR and No-DR groups was similar (46% and 43%, respectively), the CONT group had fewer (36%) male individuals. Furthermore, a group with nonproliferative diabetic retinopathy was not included, and this does not allow us to rule out the possibility of a similar increase in DNA methylation in these diabetic patients, as observed in the PDR group. However, significant differences in the DNA methylation status of the *D-Loop* region, which is responsible for encoding the majority of mtDNA-encoded genes, and promoters of two other genes important in mitochondrial function, *MLH1* and *SOD2*, in the PDR and No-DR groups strengthen the importance of DNA methylation in diabetic retinopathy.

In conclusion, ongoing research has now unraveled some associations between DNA methylation level in blood and breast cancer risk,^60^ and analysis of blood from patients enrolled in the landmark Diabetes Control and Complications Trial-Epidemiology of Diabetes Interventions and Complications Study has suggested the possible use of DNA methylation status, especially that of thioredoxin-interacting protein, as a biomarker for glycemia.^61^ Methylation of mtDNA is shown to be involved in pathological phenotypes of chronic diseases such as nonalcoholic steatohepatitis and Down's syndrome^37^ and is considered as a possible biomarker for chromium exposure.^62^ The retina from donors with age-related macular degeneration have higher levels of mtDNA deletions/rearrangements with higher single nucleotide polymorphisms in the *D-Loop*.^63^ Despite some limitations listed above, our exciting results show significantly higher DNA methylation of mtDNA and associated genes in the peripheral blood from proliferative diabetic retinopathy patients, and these results suggest a possible correlation between DNA methylation and proliferative diabetic retinopathy.

### Translational Relevance

Mitochondrial homeostasis is critical for their function, and as described above, basic research using both in vitro and in vivo models of diabetic retinopathy has shown the importance of DNA methylation in mitochondrial homeostasis. Here, using peripheral blood from patients, we show that methylation of mtDNA and that of promoter DNA of the enzymes critical in mitochondrial homeostasis is strongly correlated with the presence of diabetic retinopathy. This could open up a possibility of employing DNA methylation as a potential biomarker of retinopathy in diabetic patients and bridging the gap between basic research and clinical care. DNA methylation status in the blood of diabetic patients could also serve as an important readout parameter for testing longitudinal outcome of novel therapeutics for this blinding disease, furthering the translational impact of our study.

## References

[1] Ogurtsova K, da Rocha Fernandes JD, Huang Y (2017). IDF Diabetes atlas: global estimates for the prevalence of diabetes for 2015 and 2040. *Diab Res Clin Pract*.

[2] Frank RN (2004). Diabetic retinopathy. *New Eng J Med*.

[3] UKPDS (1998). Tight blood pressure control and risk of macrovascular and microvascular complications in type 2 diabetes: UKPDS 38. UK Prospective Diabetes Study Group. *BMJ*.

[4] Ting DS, Cheung GC, Wong TY (2016). Diabetic retinopathy: global prevalence, major risk factors, screening practices and public health challenges: a review. *Clin Exp Ophthalmol*.

[5] Lee R, Wong TY, Sabanayagam C (2015). Epidemiology of diabetic retinopathy, diabetic macular edema and related vision loss. *Eye Vis (Lond)*.

[6] Vijan S (2015). In the clinic. Type 2 diabetes. *Ann Intern Med.*.

[7] Diabetes Control and Complications Trial Research Group (1993). The effect of intensive treatment of diabetes on the development of long-term complications in insulin-dependent diabetes mellitus. *New Eng J Med*.

[8] Lachin JM, White NH, Diabetes C, Complications Trial/Epidemiology of Diabetes I, Complications Research G (2015). Effect of intensive diabetes therapy on the progression of diabetic retinopathy in patients with type 1 diabetes: 18 years of follow-up in the DCCT/EDIC. *Diabetes*.

[9] Group DER, Aiello LP, Sun W (2015). Intensive diabetes therapy and ocular surgery in type 1 diabetes. *New Eng J Med*.

[10] Kowluru RA, Kowluru A, Mishra M, Kumar B (2015). Oxidative stress and epigenetic modifications in the pathogenesis of diabetic retinopathy. *Prog Retin Eye Res*.

[11] Kowluru RA (2005). Diabetic retinopathy: mitochondrial dysfunction and retinal capillary cell death. *Antioxid Redox Signal*.

[12] Kowluru RA, Santos JM, Mishra M (2013). Epigenetic modifications and diabetic retinopathy. *Biomed Res Int*.

[13] Roy S, Trudeau K, Roy S, Tien T, Barrette KF (2013). Mitochondrial dysfunction and endoplasmic reticulum stress in diabetic retinopathy: mechanistic insights into high glucose-induced retinal cell death. *Curr Clin Pharmacol*.

[14] Kowluru RA, Mishra M (2017). Epigenetic regulation of redox signaling in diabetic retinopathy: role of Nrf2. *Free Radic Biol Med*.

[15] Feil R, Fraga MF (2012). Epigenetics and the environment: emerging patterns and implications. *Nat Rev Genet*.

[16] Pruitt K (2016). Molecular and cellular changes during cancer progression resulting from genetic and epigenetic alterations. *Prog Mol Biol Trans Sci*.

[17] Maghbooli Z, Hossein-Nezhad A, Larijani B, Amini M, Keshtkar A (2015). Global DNA methylation as a possible biomarker for diabetic retinopathy. *Diabetes Metab Res Rev*.

[18] Costantino S, Ambrosini S, Paneni F (2019). The epigenetic landscape in the cardiovascular complications of diabetes. *J Endocrinol Invest*.

[19] Lister R, Pelizzola M, Dowen RH (2009). Human DNA methylomes at base resolution show widespread epigenomic differences. *Nature*.

[20] Moore LD, Le T, Fan G (2013). DNA methylation and its basic function. *Neuropsychopharmacology*.

[21] Bergman Y, Cedar H (2013). DNA methylation dynamics in health and disease. *Nat Struct Mol Biol*.

[22] Delatte B, Deplus R, Fuks F (2014). Playing TETris with DNA modifications. *EMBO J*.

[23] Zhang P, Huang B, Xu X, Sessa WC (2013). Ten-eleven translocation (Tet) and thymine DNA glycosylase (TDG), components of the demethylation pathway, are direct targets of miRNA-29a. *Biochem Biophys Res Commun*.

[24] Bell CG, Teschendorff AE, Rakyan VK, Maxwell AP, Beck S, Savage DA (2010). Genome-wide DNA methylation analysis for diabetic nephropathy in type 1 diabetes mellitus. *BMC Med Genomics*.

[25] Maghbooli Z, Larijani B, Emamgholipour S, Amini M, Keshtkar A, Pasalar P (2014). Aberrant DNA methylation patterns in diabetic nephropathy. *J Diabetes Metab Disord*.

[26] Kowluru RA, Mishra M (2015). Oxidative stress, mitochondrial damage and diabetic retinopathy. *Biochim Biophys Acta*.

[27] Kowluru RA, Mishra M (2015). Contribution of epigenetics in diabetic retinopathy. *Sci China*. *Life Sci*.

[28] Scarpulla RC (2012). Nucleus-encoded regulators of mitochondrial function: integration of respiratory chain expression, nutrient sensing and metabolic stress. *Biochim Biophys Acta*.

[29] Stuart JA, Brown MF (2006). Mitochondrial DNA maintenance and bioenergetics. *Biochim Biophys Acta*.

[30] Clayton DA (2000). Transcription and replication of mitochondrial DNA. *Hum Reprod*.

[31] Rothfuss O, Gasser T, Patenge N (2010). Analysis of differential DNA damage in the mitochondrial genome employing a semi-long run real-time PCR approach. *Nucleic Acid Res*.

[32] Sharma H, Singh A, Sharma C, Jain SK, Singh N (2005). Mutations in the mitochondrial DNA D-loop region are frequent in cervical cancer. *Cancer Cell Int*.

[33] Mishra M, Kowluru RA (2019). DNA methylation–a potential source of mitochondria DNA base mismatch in the development of diabetic retinopathy. *Mol Neurobiol*.

[34] Branco MR, Ficz G, Reik W (2011). Uncovering the role of 5-hydroxymethylcytosine in the epigenome. *Nat Rev Genet*.

[35] Shock LS, Thakkar PV, Peterson EJ, Moran RG, Taylor SM (2011). DNA methyltransferase 1, cytosine methylation, and cytosine hydroxymethylation in mammalian mitochondria. *Proc Natl Acad Sci U S A*.

[36] Dzitoyeva S, Chen H, Manev H (2012). Effect of aging on 5-hydroxymethylcytosine in brain mitochondria. *Neurobiol Aging*.

[37] Iacobazzi V, Castegna A, Infantino V, Andria G (2013). Mitochondrial DNA methylation as a next-generation biomarker and diagnostic tool. *Mol Genet Metab*.

[38] Manev H, Dzitoyeva S, Chen H (2012). Mitochondrial DNA: a blind spot in neuroepigenetics. *Biomol Concepts*.

[39] Ji F, Zhao C, Wang B, Tang Y, Miao Z, Wang Y The role of 5-hydroxymethylcytosine in mitochondria after ischemic stroke. *J Neurosci Res.* 8;.

[40] Mishra M, Kowluru RA (2015). Epigenetic modification of mitochondrial DNA in the development of diabetic retinopathy. *Invest Ophthalmol Vis Sci*.

[41] Kowluru RA (2019). Mitochondrial stability in diabetic retinopathy: lessons learned from epigenetics. *Diabetes*.

[42] Xu Y, Li H, Hedmer M (2017). Occupational exposure to particles and mitochondrial DNA–relevance for blood pressure. *Environ Health*.

[43] Byun HM, Colicino E, Trevisi L, Fan T, Christiani DC, Baccarelli AA (2016). Effects of air pollution and blood mitochondrial dna methylation on markers of heart rate variability. *J Am Heart Assoc*.

[44] Zheng LD, Linarelli LE, Brooke J (2016). Mitochondrial epigenetic changes link to increased diabetes risk and early-stage prediabetes indicator. *Oxid Med Cell Longev*.

[45] Mishra M, Lillvis J, Seyoum B, Kowluru RA (2016). Peripheral blood mitochondrial DNA damage as a potential noninvasive biomarker of diabetic retinopathy. *Invest Ophthalmol Vis Sci*.

[46] Jensen TJ, Kim SK, Zhu Z (2015). Whole genome bisulfite sequencing of cell-free DNA and its cellular contributors uncovers placenta hypomethylated domains. *Genome Biol*.

[47] Kowluru RA, Shan Y, Mishra M (2016). Dynamic DNA methylation of matrix metalloproteinase-9 in the development of diabetic retinopathy. *Lab Invest*.

[48] Mishra M, Kowluru RA (2014). Retinal mitochondrial DNA mismatch repair in the development of diabetic retinopathy, and its continued progression after termination of hyperglycemia. *Invest Ophthalmol Vis Sci*.

[49] Zhong Q, Kowluru RA (2011). Epigenetic changes in mitochondrial superoxide dismutase in the retina and the development of diabetic retinopathy. *Diabetes*.

[50] Keating ST, van Diepen JA, Riksen NP, El-Osta A (2018). Epigenetics in diabetic nephropathy, immunity and metabolism. *Diabetologia*.

[51] Nicholls TJ, Minczuk M (2014). In D-loop: 40 years of mitochondrial 7S DNA. *Exp Gerontol*.

[52] Fish J, Raule N, Attardi G (2004). Discovery of a major D-loop replication origin reveals two modes of human mtDNA synthesis. *Science*.

[53] Stoneking M (2000). Hypervariable sites in the mtDNA control region are mutational hotspots. *Am J Hum Genet*.

[54] Ehrlich M, Norris KF, Wang RY, Kuo KC, Gehrke CW (1986). DNA cytosine methylation and heat-induced deamination. *Biosci Rep*.

[55] Martin SA, McCabe N, Mullarkey M (2010). DNA polymerases as potential therapeutic targets for cancers deficient in the DNA mismatch repair proteins MSH2 or MLH1. *Cancer Cell*.

[56] Madsen-Bouterse SA, Mohammad G, Kanwar M, Kowluru RA (2010). Role of mitochondrial DNA damage in the development of diabetic retinopathy, and the metabolic memory phenomenon associated with its progression. *Antioxid Redox Signal*.

[57] Azadmanesh J, Borgstahl GEO (2018). A review of the catalytic mechanism of human manganese superoxide dismutase. *Antioxidants*.

[58] Cyr AR, Hitchler MJ, Domann FE (2013). Regulation of SOD2 in cancer by histone modifications and CpG methylation: closing the loop between redox biology and epigenetics. *Antioxid Redox Signal*.

[59] Ozawa GY, Bearse MA, Adams AJ (2015). Male-female differences in diabetic retinopathy?. *Curr Eye Res*.

[60] Tang Q, Cheng J, Cao X, Surowy H, Burwinkel B (2016). Blood-based DNA methylation as biomarker for breast cancer: a systematic review. *Clin Epigenetics*.

[61] Chen Z, Miao F, Paterson AD (2016). Epigenomic profiling reveals an association between persistence of DNA methylation and metabolic memory in the DCCT/EDIC type 1 diabetes cohort. *Proc Natl Acad Sci U S A*.

[62] Yang L, Xia B, Yang X (2016). Mitochondrial DNA hypomethylation in chrome plating workers. *Toxicol Lett*.

[63] Kenney MC, Atilano SR, Boyer D (2010). Characterization of retinal and blood mitochondrial DNA from age-related macular degeneration patients. *Invest Ophthalmol Vis Sci*.

